# HIV Prevention and Treatment Interventions for Black Men Who Have Sex With Men in Canada: Scoping Systematic Review

**DOI:** 10.2196/40493

**Published:** 2024-01-18

**Authors:** Jemal Demeke, Pascal Djiadeu, Abban Yusuf, Darren Lovell Whitfield, David Lightfoot, Fiqir Worku, Gamji Rabiu Abu-Ba'are, Lawrence Mbuagbaw, Sulaimon Giwa, LaRon E Nelson

**Affiliations:** 1 MAP Centre for Urban Health Solutions Li Ka Shing Knowledge Institute St Michael’s Hospital, Unity Health Toronto Toronto, ON Canada; 2 Department of Health Research Methods, Evidence and Impact McMaster University Hamilton, ON Canada; 3 School of Social Work University of Maryland Baltimore, MD United States; 4 Institute of Health Policy, Management, and Evaluation University of Toronto Toronto, ON Canada; 5 Center for Interdisciplinary Research on AIDS Yale School of Public Health New Haven, CT United States; 6 School of Social Work St John's College Memorial University of Newfoundland St John's, NL Canada; 7 School of Nursing Yale University New Haven, CT United States

**Keywords:** men who have sex with men, African, Caribbean, Black, HIV and AIDS, epidemiology, public health, HIV, health care, prevention

## Abstract

**Background:**

Black men who have sex with men (MSM) experience disproportionately high HIV incidence globally. A comprehensive, intersectional approach (race, gender, and sexuality or sexual behavior) in understanding the experiences of Black MSM in Canada along the HIV prevention and care continuums has yet to be explored.

**Objective:**

This scoping review aims to examine the available evidence on the access, quality, gaps, facilitators, and barriers of engagement and identify interventions relevant to the HIV prevention and care continuum for Black MSM in Canada.

**Methods:**

We conducted a systematic database search, in accordance with the PRISMA-ScR (Preferred Reporting Items for Systematic Reviews and Meta-Analyses extension for Scoping Reviews) checklist, of the available studies on HIV health experience and epidemiology concerning Black MSM living with or without HIV in Canada and were published after 1983 in either English or French. Searched databases include MEDLINE, Excerpta, Cumulative Index to Nursing and Allied Health Literature, the Cochrane Library, the NHUS Economic Development Database, Global Health, PsycInfo, PubMed, Scopus, and Web of Science. From the 3095 articles identified, 19 met the inclusion criteria and were analyzed.

**Results:**

Black MSM in Canada consistently report multiple forms of stigma and lack of community support contributing to an increased HIV burden. They experience discrimination based on their intersectional identities while accessing HIV preventative and treatment interventions. Available data demonstrate that Black MSM have higher HIV incidences than Black men who have sex with women (MSW) and White MSM, and low preexposure prophylaxis knowledge and HIV literacy. Black MSM experience significant disparities in HIV prevention and care knowledge, access, and use. Structural barriers, including anti-Black racism, homophobia, and xenophobia, are responsible for gaps in HIV prevention and care continuums, poor quality of care and linkage to HIV services, as well as a higher incidence of HIV.

**Conclusions:**

Considering the lack of targeted interventions, there is a clear need for interventions that reduce HIV diagnoses among Black MSM, increase access and reduce structural barriers that significantly affect the ability of Black MSM to engage with HIV prevention and care, and address provider’s capacity for care and the structural barriers. These findings can inform future interventions, programming, and tools that may alleviate this HIV inequity.

**International Registered Report Identifier (IRRID):**

RR2-10.1136/bmjopen-2020-043055

## Introduction

The Canadian publicly funded health care system aims to provide universal access to medically necessary services [1]. This equitable goal alludes to Canadian values of equity and fairness in the administration of health and social services and serves as a source of collective pride for many Canadians [1,2]. However, this narrative does not reflect the state of access to Canadian health care services and the poor health outcomes experienced by marginalized populations [3-10]. These gaps are demonstrated through the HIV inequities seen in Black (descendants of Africa, African diaspora communities in Canada and the Caribbean) communities in Canada. For example, although Black Canadians comprise less than 4% of the national population, according to the 2017 HIV Surveillance report, they made up 25% of new HIV diagnoses [11]. In Ontario alone, between 2012 and 2017, the proportion of new HIV diagnoses increased for Black men while it decreased for White men [12]. Moreover, HIV exposure for gay, bisexual, and other men who have sex with men (MSM) represents the most significant proportion of reported adult HIV diagnoses [11]. Black MSM exist at the intersection of these 2 populations that demonstrate disproportionately high HIV incidence in Canada. Thus, sexual behavior and racialization are significant factors to HIV transmission in adult male HIV diagnoses; this illustrates the relationships between social identities and HIV health inequities in Canada.

Black MSM experience systemic anti-Black racism in addition to the sexual minority stigma and homophobic discrimination [13]. As other high-income countries work to address these inequities, it becomes increasingly important to identify the drivers of HIV inequity through the intersectional experiences of Black MSM in Canada and develop effective evidence-informed intervention models [14-16]. Unfortunately, HIV surveillance by Canadian federal and provincial health agencies does not provide the necessary disaggregated data to elucidate HIV transmission patterns for Black MSM fully. For example, only half of the Public Health Agency of Canada reported cases of integrated exposure category and race data in 2016 and 2017, while the 2018 report did not report this [11,17,18]. Even so, the limited available data reveal that Black MSM are overrepresented in the new HIV diagnoses [11]. Canada aimed to meet the Joint United Nations Programme on HIV/AIDS global targets by 2020 and significantly reduce HIV transmissions by 2030; to achieve this goal, Canada must develop a clear strategy to respond to the increased burden of HIV for Black MSM.

Socially constructed identities, including assignment to racial and sexual minority or gendered groups, are associated with inequitable and disproportionate HIV infections [19]. The factors that lead to poor health outcomes are linked to intersectional and systemic barriers, including inaccessible health care services for Black MSM. Additional factors driving poor health outcomes for Black MSM include precarious employment and housing, stress-related mental health challenges, low sexual health and HIV literacy, as well as poor psychosocial health and general health [20-22]. These factors also limit the ability of Black MSM to meaningfully and consistently engage with HIV health care services [23]. Moreover, Black MSM are exposed to anti-Black racism and homophobia within health care institutions, resulting in psychological trauma and medical trauma [13,21,24-27]. Thus, Black MSM are ultimately prevented from accessing HIV-related services by inequitable care, discrimination in health care settings, and systemic marginalization [13,23]. Yet, as these powerful barriers persist, evidence demonstrates that multilevel interventions have successfully mitigated HIV inequity by improving the accessibility to and quality of health care services [24,28,29].

The current HIV prevention and treatment interventions are considered as a continuum of services [30]. This provides a framework through which key points in prevention, care, and existing gaps are engaged [30]. Specifically, the HIV prevention, engagement, and care cascade models the steps to prevent HIV infection or receive treatment if diagnosed with HIV [30-32]. This cascade can be used to identify the gaps in HIV prevention and care and the necessary interventions. However, Canadian HIV surveillance and response efforts quantify the state of HIV transmission and service access by compartmentalizing gender, sexual minority status and sexuality, and race as individual factors. This approach does not accurately characterize the intersectional marginalization of Black MSM. Intersectionality, coined by Crenshaw [33] in 1989, established that singular identities could not explain the complex forms of oppression experienced by marginalized peoples.

Similarly, in the context of Black MSM, their experiences must investigate their gender, sexuality, and race holistically. This may then inform HIV prevention and care interventions and strategies to reduce HIV incidence for Black MSM effectively. This intersectional approach to HIV research, however, has not been widely examined [34]. The results from such investigation could promote access to and improve the quality of prevention and care along the continuums for Black MSM in Canada. Nonetheless, the limited availability of HIV interventions, research, and literature for Black MSM in Canada necessitated this investigation into past and recent HIV research as well as health care provision of HIV-related services for this vulnerable population.

This scoping review examines the state of HIV-related research and health care provision for Black MSM in Canada. Overall, this review aims to comprehensively evaluate and determine the state of HIV prevention and care for Black MSM in Canada as a public health topic while incorporating the lived experience of Black MSM that influence health care engagement. The primary objective is to assess the available literature regarding the influence, access to, and quality of HIV prevention and treatment for Black MSM living in Canada. Secondary objectives are to explore the facilitators and disincentives or barriers to HIV-related health care services for Black MSM and the mechanisms through which they influence retention and adherence to the HIV care continuum. Although this review is focused on Black MSM in Canada, its findings will be useful for ethnic minority MSM anywhere in the world.

## Methods

### Overview

The current investigation used a scoping systematic review methodology. Peterson et al [35] identified this approach as a methodology to advance emerging research topics. Standardized systematic reviews and scoping reviews differ in terms of the specificity of their topic of interest. Scoping reviews tend to investigate broad topics compared to standardized, systematic reviews, which examine a specific, detailed question.

### Criteria for Including Studies

This review included evidence syntheses and qualitative, experimental (randomized or nonrandomized), observational (longitudinal and cross-sectional), and mixed methods studies.

For inclusion in the review, studies must (1) include data on self-identified Black MSM living with or without HIV, (2) research HIV prevention and care among Black MSM, and (3) have been published in at least 1 of the 2 official languages in Canada—English and French.

For exclusion in the review, studies must not (1) focus on Black MSM outside of Canada unless Canadian data are analyzed separately and (2) be published before the 1983 formation of Canada’s AIDS National Task Force.

### Objectives of Interest

The primary objectives of interest of this scoping, systematic review include (1) identifying existing literature that examines the HIV prevention and care continuum in Canada for Black MSM, including epidemiological trends, determinants of engagement, and health care experience and (2) determining health access and availability of specific health resources related to the Canadian HIV prevention and treatment interventions for Black MSM.

The secondary objectives of interest include (1) examining the effects of social determinants of health on the HIV prevention and treatment interventions for Black MSM in Canada, (2) consolidating research on unilevel and multilevel interventions that address the social determinants of health for Black MSM in Canada, and (3) identifying existing health promotion for Black MSM in Canada.

### Patients and Public Involvement

There was no direct involvement of the public and patients or participants who are Black MSM in the study.

### Ethical Considerations

The approval of the Research Ethics Board was not required. As a scoping systematic review, information was based on secondary published data and not on human subjects. Peer-reviewed manuscripts, conference presentations, and students’ rounds can be strategically used to disseminate study findings. The findings may also influence policy within government health agencies and local HIV or AIDS service organizations.

### Search Strategy for Identification of Studies

A health sciences librarian conducted a comprehensive literature search of studies published through the Health Sciences Library in Toronto, Ontario’s St Michael’s Hospital, Unity Health Toronto. This occurred in April 2020. The list of our search terms included “Quality of Health Care,” “Health Status Disparities,” “Social Stigma,” “Human Immunodeficiency Virus,” “Same-Sex Intercourse,” “Blacks OR African OR Caribbean.” The complete search strategy can be found in the published protocol [36].

### Electronic Searches

These searches were done on MEDLINE, Excerpta, Cumulative Index to Nursing and Allied Health Literature, the Cochrane Library, the NHUS Economic Development Database, Global Health, PsycInfo, PubMed, Scopus, and Web of Science.

### Reference Lists

Related articles were searched for in the reference lists of all pertinent citations.

### Gray Literature

Available thesis and conference posters were searched while reports from relevant organizations such as the African and Caribbean Council on HIV or AIDS in Ontario (ACCHO), Black Coalition for AIDS Prevention (Black CAP), Africans in Partnership Against AIDS (APAA), Committee for Accessible AIDS Treatment (CAAT), TAIBU Community Health Centre, Ontario HIV Treatment Network (OHTN), and Canada's source for HIV and hepatitis C information were explored.

### Screening

The studies were deduplicated in advance, and the web-based app Rayyan QCRI (Rayyan) was used to import and screen citations found using this search strategy [37].

A data collection form customized to reflect the inclusion criteria was pilot tested by 2 independent reviewers. These reviewers played a role in generating and using the given form. A total of 50 abstracts were used as a sample to create consistency of use and establish the instrument’s validity. Interrater reliability was measured using the Cohen κ statistic. The screening began upon the achievement of a 60% (n=979) agreement [38].

The study’s selection process began with title and abstract screening to identify potentially relevant articles. This was followed by retrieving the full text for detailed screening using the inclusion and exclusion criteria before the data extraction procedure. All screening and data extraction were completed in duplicate and blinded by JD, PD, FW, AY, and GRAB. Any disagreements were settled via consensus. However, a third author was available to arbitrate (SG) if an agreement could not be reached.

### Data Extraction

Bibliometric information such as author names, journal, year of publication, the study location, design, number of participants, outcomes reported, outcome measures overall, and outcome measures in Black MSM participants were extracted. In addition, each outcome is reported through measures of magnitude mean (SD) or percent (95% CIs) where possible, comparing the effect of the intervention in Black MSM versus men in other racialized groups or Black MSW; (odds or risk ratios, mean differences, accompanied with 95% CIs) [39].

### Assessment of Methodological Quality of the Included Studies

We did not appraise the methodological quality and risk of bias in the studies as this is not required in a scoping review [40].

### Analyses and Reporting

Study findings were reported as per the PRISMA-ScR (Preferred Reporting Items for Systematic Reviews and Meta-Analyses extension for Scoping Reviews) guidelines [41,42] through the use of narratives and tables. This is outlined in Multimedia Appendix 1. Data were grouped according to outcomes, with the number of studies and their design displayed using tables. In addition, a narrative synthesis of the data was conducted to identify overlapping themes and knowledge gaps.

## Results

### Results of the Search

#### Overview

**Figure 1:**
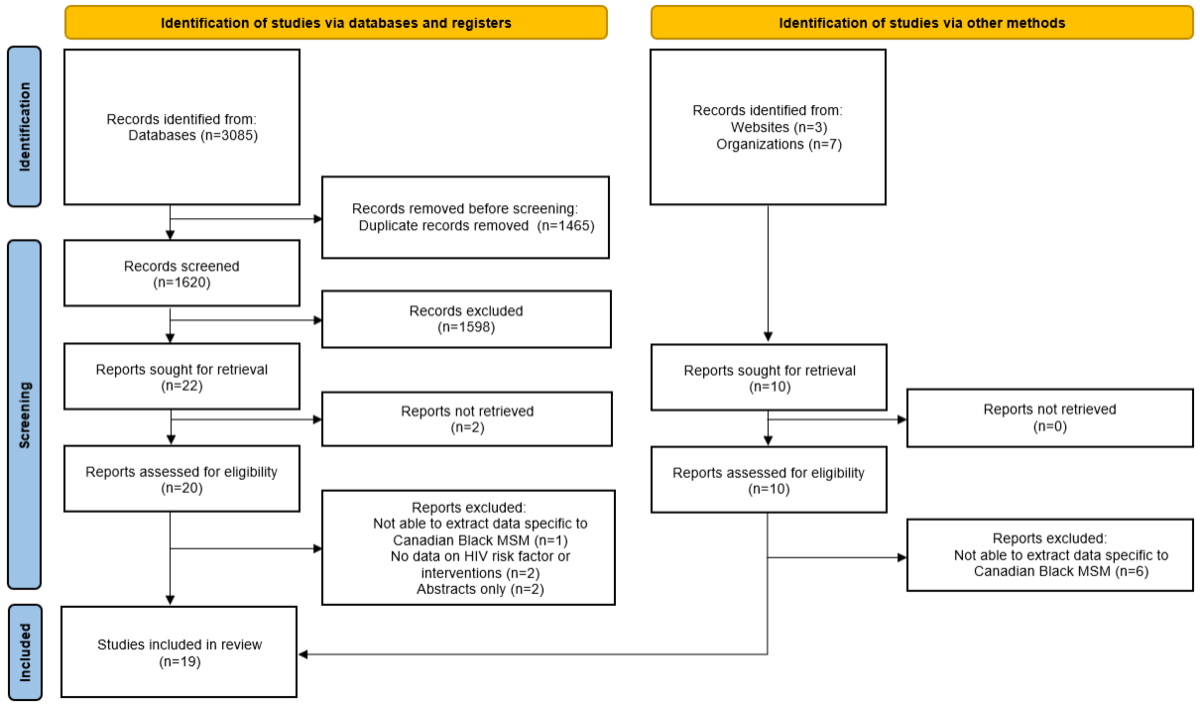
PRISMA flowchart. MSM: men who have sex with men; PRISMA: Preferred Reporting Items for Systematic Reviews and Meta-Analyses.

The literature search of the database identified 3085 studies, with 10 studies found through gray literature and other sources (Figure 1). Thus, a total of 1630 studies were screened after the duplicates were removed. A total of 1607 studies were excluded as they did not study Black MSM in Canada or did not include any data on HIV prevention and treatment interventions. Of the remaining 24 studies, 4 were removed during full-text extraction; among them, 2 were abstracts, 1 study reported Canadian and American Black MSM together, and the other 2 did not provide information on HIV. This left 19 studies that met the inclusion criteria and were included in the study.

#### Characteristics of the Study

There were 8 cross-sectional descriptive studies, 3 qualitative studies, 2 mixed methods studies, 1 meta-analysis, and 5 organizational reports. The studies’ publication years ranged from 2004 to 2020. The study characteristics are summarized in Table 1. Further description of each study is provided in Multimedia Appendix 2.

**Table 1:** Summary of results by themes and number of studies included.

Theme or subtheme		Studies included, n
**Engagement with HIV prevention and care cascades**		
	HIV epidemiology and its driving factors	4
	Factors that influence HIV testing	7
	Preexposure prophylaxis use among Black MSM^a^	2
**Experiences in health care for Black MSM**		
	Discrimination in HIV health care	3
	Inadequate HIV health care provision	2
	Missed opportunities for linkage to HIV prevention and care	3
**Social determinants of health and HIV health care access**		
	Stigma and homophobia	6
	Employment, income, and housing	6
	Immigration	3
	Education and access to knowledge about HIV prevention and intervention methods	3
	Mental health and emotional well-being	6
**Canadian HIV interventions for Black MSM**		
	Biomedical interventions	5
	Behavioral interventions	5
	Structural interventions	1

^a^MSM: men who have sex with men.

#### Reporting of Results

The results of the studies included in the scoping systematic review can be grouped into 4 themes identified from the data and are discussed below. They are the engagement with HIV prevention and care cascades, experiences in HIV health care services, the effects of the social determinants of health on HIV prevention and care access, and interventions for Black MSM.

### Engagement With HIV Prevention and Care Cascades

#### Overview

A total of 10 studies explored the engagement of Black MSM with the various steps in the HIV prevention and care continuum.

#### HIV Epidemiology and its Driving Factors

Multiple studies assessed HIV infections for Black MSM and their predictors. In Ontario, Black MSM reported the highest increase of HIV diagnoses among racialized MSM, compared to the decreasing HIV diagnoses for White MSM over 2009-2010 and 2011-2012 [43]. This disproportionate increase for Black MSM is supported in other studies in this review [44,45].

Moreover, 2 studies explored differences in HIV diagnoses and associated factors between Black MSW and Black MSM in Toronto. Black MSM demonstrated considerably higher HIV and syphilis prevalence compared to Black MSW [44,46]. Significant HIV diagnosis predictors were the number of male partners (6 or higher), older than 24 years of age, and syphilis diagnoses [44,46]. In addition, Black MSM showed higher transmission of sexual and blood-borne infections (SBBIs) as well, including chlamydia, herpes simplex virus (HSV) 1, HSV-2, and active hepatitis B (HBV) infection compared to other MSM [44,45].

#### Factors That Influence HIV Testing

HIV testing behaviors among Black MSM were also investigated. Generally, African, Caribbean, and Black MSM had lower HIV testing rates than other ethnicities [43,47]. Straight-identified Black MSM were more likely to have had an HIV test than gay-identified Black MSM [45]. Canadian-born Black MSM were less likely to get tested for HIV [48].

Several studies assessed factors that influence HIV testing for Black MSM. Black MSM were shown to have a stronger likelihood of being tested for HIV if they were older than 21 years of age, engaged in condomless sex, had a history of a chlamydia infection, or had relatives or friends who live with or died from HIV [44,48]. Caribbean- and African-born MSM were more likely to know someone who currently lives with HIV or know someone who died from complications due to HIV than Canadian-born Black MSM [49,50]. Nontesters commonly cite low perception of risk and practicing safe sex as reasons for not testing for HIV [48]. Overall, Black MSM are most likely to be tested through their family doctor [48].

#### Preexposure Prophylaxis Use Among Black MSM

It is important to highlight that preexposure prophylaxis (PrEP) is a highly effective oral antiretroviral medication used in the prevention of HIV [51]. While the implementation of PrEP through the Canadian guidelines remains controversial due to concerns about accessibility for Black populations, PrEP is identified as a promising tool to reduce HIV incidence in Canada [52,53].

This review finds that similar to the trends seen with HIV testing, Black MSM in younger age groups and Canadian-born were less likely to accept PrEP [54]. However, Black MSM were found to accept PrEP at greater rates than Black MSW and similar rates to other MSM populations [54]. As a whole, PrEP acceptance was not significantly associated with self–risk perception [54]. More than half of Black MSM could not accurately describe their actual risk for HIV acquisition [54]. For those who did not accept PrEP, the most popular explanations for not accepting PrEP were concerns about side effects and low perception of risk for HIV transmission [54,55].

### Experiences in Health Care for Black MSM

#### Overview

A total of 4 studies assessed the specific experiences that Black MSM have while accessing HIV health care services in Canada and the quality of care in the context of their intersectional experiences as Black MSM.

#### Discrimination in HIV Health Care

Black MSM consistently reported discrimination and inequitable access to HIV care and preventative interventions like PrEP [55-57]. In general, Black MSM noticed that health care staff neglected them in favor of White patients [55,56]. Black MSM who self-identify as gay, bisexual, transgender, or queer (GBTQ+) felt that their Blackness remained a barrier to care [55,57]. Furthermore, upon entering Black-centered health service organizations, many Black MSM often did not feel comfortable disclosing their sexual practices [55,57].

#### Inadequate HIV Health Care Provision

Black MSM describe low-quality and uncompassionate care, which facilitates gaps in HIV care and disrupts retention of care [56]. In addition, health care providers often use scientific or medical terms that are too complex [56]. This contributes to a more prominent theme of depersonalized care, where health care providers appear neither genuine nor have investments in their sexual health [55,56]. Moreover, mental health and emotional well-being were not incorporated into the HIV care provided to Black MSM, especially when health care providers were disclosing HIV diagnoses [56].

#### Missed Opportunities for Linkage to HIV Prevention and Care

Health care providers fail to inform Black MSM about the HIV resources and the prevention and treatment interventions that they could access, despite health care providers being described as a key source of sexual health information [55,58]. Black MSM needed to advocate for their care in response to the negligence of their health care providers [55,56]. GBTQ+ Black MSM who seek sexual health services from queer-friendly providers often have long wait times for services, which affects HIV care adherence [55]. Furthermore, many Black MSM did not know about PrEP or had inaccurate knowledge about PrEP despite regular HIV testing [55].

### Social Determinants of Health and HIV Health Care Access

#### Overview

A total of 10 studies reported various social factors as facilitators and barriers to HIV care and prevention services for Black MSM.

#### Stigma and Homophobia

HIV stigma and sexual minority stigma are significant stressors for Black MSM and impair HIV prevention and care access and engagement [56,59]. Also, Black MSM living with HIV fear stigmatization and rejection can reduce their likelihood of disclosing their HIV status to their sexual partners [56]. GBQT+ Black MSM have an even increased stigma burden due to homophobia [45,57,58]. The fear of ostracization from their communities also prevents Black MSM from talking about their sexual practices and HIV as a whole [57]. HIV stigma and sexual minority stigma also affect their perception of risk for HIV acquisition [59]. Specifically, heterosexual-identified Black MSM internalize messaging that associates HIV risk with being gay, compromising their own perception of risk [56,57]. For instance, “PrEP stigma” is prevalent as many Black MSM associate PrEP with promiscuity [59]. This stigma translates to increased concerns about PrEP and decreased knowledge about PrEP [59].

Regardless of sexual identity, these stigmas make Black MSM apprehensive about disclosing their sexual practices and deter them from accessing critical HIV interventions [45,55,56,58]. This limits the providers’ ability to inform Black MSM about HIV prevention interventions usually promoted to MSM [55]. Black MSM are also concerned about judgment upon entering HIV health care organizations or having their privacy and confidentiality breached [45,56].

#### Employment, Income, and Housing

Black MSM experience hardship securing full-time and part-time work, and many report unemployment as a priority concern [50,57]. Black MSM in the MaBwana study had lower incomes than the average income of a sample of MSM living in Ontario [50]. This may contribute to difficulties with securing housing and poverty [57]. These disparities affect their use of HIV prevention interventions such as PrEP, which is the most significant biomedical HIV prevention intervention [49,60]. The cost was described as a significant barrier to PrEP use—even if Black MSM were willing to accept PrEP, they report that its unaffordability prevents them from discussing PrEP with their health care providers [55,59]. Nonetheless, while there are various ways to access PrEP through public and private insurance, many Black MSM are unaware of PrEP coverage to mitigate PrEP costs [55,59].

#### Immigration

Migrants have distinct experiences in accessing HIV prevention and treatment interventions. While many Black MSM favor Canadian health care, they also describe language barriers and stress with navigating complex immigration laws [56]. Without having the necessary immigration documentation, some Black MSM have been denied medication and HIV care [56]. Many Black MSM born outside of Canada have low HIV prevention literacy and have experienced medical trauma [58]. Also, they may doubt the efficacy of PrEP by emphasizing its adverse effects [58]. Moreover, migrant GBTQ+ Black MSM face anti-Black racism and xenophobia in White gay communities, where more people may be using or know about PrEP [50]. This leaves many migrant Black MSM without the HIV and PrEP knowledge other communities may have [50].

#### Education and Access to Knowledge About HIV Prevention and Intervention Methods

The MaBwana study demonstrated lower achievement of formal education for Black MSM than other MSM [50]. In addition, some Black MSM disclose low reading and writing skills, which limit their ability to learn about important sexual health and HIV information and interventions [56]. Even when they can meaningfully engage with educational institutions, Black MSM describe heteronormative sexual education and a significant lack of GBTQ+ representation [58]. Thus, they do not learn about biomedical HIV interventions and prevention methods relevant to their sexual practices [58].

#### Mental Health and Emotional Well-Being

The stigmatization of the sexual practices of Black MSM by their cultural communities has a considerable effect on their mental health [57]. This ostracization can lead Black MSM to experience feelings of isolation, alienation, and internalized homophobia. It also contributes to higher levels of depression and psychological distress [49]. These factors can impair the ability of Black MSM to access HIV treatment and care [45,50,57,61,62]. These dynamics act as barriers for Black MSM to meaningfully engage with HIV biomedical preventative interventions and mitigate the transmission of HIV [49]. Furthermore, research on Black MSM in the United States has shown that the intersection of discrimination experiences related to race or ethnicity, sexual orientation, and HIV status can significantly mediate the relation between socioeconomic status and mental health concerns [63]. These findings suggest that having low socioeconomic resources could increase exposure to discrimination and worsen mental health.

### Canadian HIV Interventions for Black MSM

#### Biomedical Interventions

Condoms remain the most accessible biomedical intervention for Black MSM [49,50,55]. Initiatives that promote condom use in settings where Black MSM engage in unplanned sex or with casual sex partners may increase condom accessibility [49,50]. The Black CAP provided condoms through their bathhouse outreach program and similar programs in other places frequented by Black MSM like clubs, barbershops, Pride, and other community events [64]. The Black CAP developed a partnership with a local sexual health clinic, Hassle-Free Clinic, to implement programming that provided quick and easy access to HIV and syphilis testing for Black MSM [65].

#### Behavioral Interventions

Several behavioral interventions identified through these studies aim to improve risk perception and HIV literacy, promote HIV prevention services, and adapt sexual behaviors to mitigate HIV transmission [49,50,59]. For example, a workshop was provided to Black-Canadian MSM and focused on increasing knowledge of sexually transmitted infections and factors contributing to their transmission and promoting sexual health service use [59]. The ACCHO campaign, “Keep It Alive,” was a multimedia initiative in 2006 that used postcards, posters, and popular gay media to increase HIV literacy and knowledge of HIV in Black communities, promote testing, and reduce HIV stigma. Likewise, the “Be Real” campaign by the Ontario Gay Men’s Strategy in 2006 had similar aims as “Keep It Alive” and used related dissemination methods. Black MSM described both campaigns as significant in providing information about sexual behavior habits and reducing the risk for HIV transmission [50].

Black CAP’s MSM Outreach program focuses on African, Caribbean, and Black MSM and aims to increase HIV awareness, HIV testing rates, and access to HIV resources and services [66]. They facilitate regular group discussions and sexual health education for Black MSM. This expands on their bathhouse outreach program, where they continue to distribute sexual health materials in bathhouses and Pride events as well as in webpages designed for GBTQ+ Black MSM. Moreover, Black CAP’s Men’s Prevention Program provides culturally responsive HIV-focused workshops to Black MSM and includes homophobia, community support, and self-esteem. Also, they developed a resource to promote PrEP knowledge, use, and access for Black MSM in Toronto [65].

#### Structural Interventions

Black CAP reported initiatives that aim to reduce social inequity and other driving factors for HIV transmission for Black MSM [65]. For example, they linked Black MSM with support services that increased access to food, housing, income, and immigration aid. They also created an employment program, “Be Your Own Boss Series,” which educates Black trans-MSM on entrepreneurship and supports developing skills to develop and maintain a successful business.

## Discussion

### Principal Findings

Overall, the purpose of this study was to evaluate engagement with HIV services and interventions, identify the relevant facilitators and barriers via the social determinants of health, and determine the research and available interventions for Black MSM in the HIV prevention and care cascade through the lens of intersectional, overlapping identities. This scoping review presents a comprehensive evaluation of the state of the science as it pertains to HIV prevention and treatment interventions for Black MSM in Canada through available literature and reports. Through the 19 published studies and reports included, this review has identified critical points in the HIV prevention and care cascades, where Black MSM experience poor outcomes in knowledge, access, engagement, and quality of HIV care and prevention.

PrEP remains the most accessible and well-established biomedical intervention available in Canada. Yet, there were no identified PrEP delivery strategies that target Black MSM in this study. To inclusively reduce HIV incidence in Canada, it is crucial to identify the factors that promote PrEP access and use. Motivations to use these prevention methods are characterized by the perception of HIV risk, adequate knowledge of the intervention and its benefits, and acceptability within the relevant sociocultural contexts for Black MSM. Limited discussion on HIV risk and prevention methods with health care providers contributes to the low-risk perception and PrEP motivation [55,56,58]. For instance, some health care providers refuse to discuss PrEP with their patients due to being uncomfortable discussing sex, sexuality, and sexual health with same-gender-loving patients [67,68]. Moreover, social stigmas including homophobia, racism, and xenophobia may affect motivation to seek HIV preventative services. These experiences can prevent Black MSM from disclosing their sexual practices, which providers need to assess HIV risk. Ultimately, these gaps expose the need for timely interventions for Black MSM [55,56,58]; priority areas include promoting the relevance of PrEP to health care providers, highlighting culturally responsive strategies, and generating space spaces actively for Black MSM to engage with providers honestly.

Access in the prevention cascade is influenced by numerous factors, including income, employment, proximity to health care facilities, housing, and other social determinants of health [64,69,70]. Many Black MSM describe precarious work and housing statuses, low income, issues related to poverty, and difficulty accessing formal education [50,57]. These disparities are associated with inadequate access to HIV prevention interventions, as high PrEP costs consistently reduce PrEP uptake [49,55,59]. More research is necessary to fully elucidate the mechanisms through which key factors influence health care engagement such as income, housing, and mental health and emotional well-being [71,72]. In the literature, MSM living with HIV experienced depression associated with physical, educational, social, financial, psychological, and short- and long-term health consequences [69]. People living with HIV who experienced depression are exposed to poor health outcomes like poor quality of life and worsening of their disease states. This could have even more consequences on Black MSM who are already impacted by other structural factors [70]. Therefore, if depression is untreated in MSM living with HIV, this can lead to risky sexual behavior, alcohol, and drug misuse and abuse, including suicide [73]. Poor adherence to antiretroviral drugs has also been associated with depression in newly diagnosed people living with HIV, resulting in poor immunological and virological outcomes.

To reduce HIV incidence for Black MSM, interventions that increase access might be beneficial including mass distribution and outreach programs, integrated health services, or legal changes that empower Black MSM to engage with HIV prevention interventions [74]. There is still limited research on intervention strategies to increase access to prevention interventions for Black MSM across Canada. Similar research in the United States investigated specific legislative and structural opportunities for Black MSM to successfully engage in HIV prevention [75,76]. Canada should commit to adopting effective programs in other countries or funding Canadian research that uses implementation science to address the identified challenges in this review for Black MSM.

While HIV prevention and care services exist along the same continuum, HIV care differs from the prevention cascade. People who remain at risk and test negative for HIV need to be linked to prevention services, but an HIV diagnosis requires a specific set of services to maintain viral suppression. The HIV care cascade models HIV testing and diagnosis steps as the starting point to viral suppression through a series of HIV health care services [30]. In comparison to other MSM, Black MSM demonstrate lower testing rates [43,47]. Still, this review shows various HIV testing behaviors among Black MSM dependent on age, sexuality, country of birth, religious affiliation, history of SBBI, and even personal relationships that have been affected by HIV [44,48-50]. Fortunately, this can inform the generation of testing interventions targeting Black MSM subgroups. For instance, many Black MSM were found to receive HIV testing from their family doctor, which can be used to build programming to link Black MSM to HIV tests directly from their primary care physician [48]. Outside of Canada, there have been many HIV testing interventions for Black MSM and other priority populations that have improved HIV testing and knowledge, testing access, and social support [77-81].

Moreover, HIV diagnosis rates are higher among Black MSM than other MSM and Black MSW [43-45]. Once diagnosed with HIV, Black MSM must be linked to HIV primary and specialist care services and antiretroviral therapy to achieve viral suppression [30]. While there were no studies on engagement at these stages by Black MSM, studies address the discriminatory barriers to access for Black MSM [55-57]. Nevertheless, the use of HIV care interventions is shown to be effective with other populations through improvements in retention of care, engagement with providers, and even condom use [82-85]. This review did not identify any HIV care interventions for Black MSM, which exposes the health care gap for Black MSM despite the epidemiological significance. Canada’s goal to dramatically reduce HIV incidence by 2030 is unachievable unless Black MSM are explicitly highlighted by service agencies.

The unilevel interventions mentioned in this study were reported in organizational reports. These are Black community–based initiatives driven by Black-focused organizations such as ACCHO, Black CAP, and APAA. Although they have strong engagement and trust with Black communities, they are limited by a lack of research to support their ability to scale up including a focus on their effectiveness, feasibility, appropriateness, and vitality. Health care organizations, government, and research funding agencies need to invest in interventions led by community stakeholders to reduce HIV incidence in Black MSM. Black MSM in Canada are entitled to an equitable standard of care and should not be confined to select organizations outside of provincially mandated health service providers.

This review provides information that can be used to develop many interventions, including programs that provide support for mental health, immigration, housing, and employment; train providers to deliver culturally responsive HIV prevention and care services; and provide accessible and affordable HIV testing and PrEP services. Combining systems-level, provider-level, and patient-level interventions may be necessary for reducing the effect of these factors, as evidence suggests these interventions improve HIV-related outcomes in Black and nonheteronormative communities [86-88]. Targeting critical points in the HIV prevention and care continuum may lead to improvements for Black MSM. Thus, there is a strong need for unilevel and multilevel HIV prevention and treatment interventions as well as more comprehensive clinical research studies that investigate the outcomes of these interventions across Canada for Black MSM.

### Limitations

The findings of this scoping review are limited by the fact that all study interventions were conducted in the Greater Toronto Area, with English as the dominant language. This approach may not be practical for capturing the diverse experiences of Black MSM across Ontario and in other Canadian provinces and territories. In addition, as no French studies were identified in this review, the specific barriers that Francophone Black MSM experience may not be included.

### Conclusions

MSM and Black communities are priority populations most affected by HIV in Canada. Black MSM experience disparities in HIV incidence and are disproportionately affected by the driving factors of HIV transmission, including stigma and discrimination. Singular-focused MSM or Black-specific interventions may not be effective for Black MSM, as they need intersectional intervention strategies that affirm their racial and sexual identities and sexual practices. This review demonstrates evidence that Black MSM experience structural discrimination, stigma, and poor quality of care and linkage to HIV services, and as a result, higher HIV and other SBBIs impact. There is some evidence of HIV interventions in Canada for Black MSM. Still, none of the interventions identified in this scoping review were assessed by randomized control trials to evaluate their effectiveness in alleviating HIV transmission disparities for Black MSM. Thus, an important next step is to develop evidence-based HIV interventions to reduce HIV inequities for Black MSM in Canada. The benefit of this review is that it provides a comprehensive overview of the barriers for Black MSM in HIV care from the individual to environmental, institutional, and structural. Additionally, this review has highlighted gaps in care that need to be addressed and sheds light on the intersectional interplay of identities in Black MSM care. This review can inform the development of these interventions for Black MSM and outline the factors that need to be addressed. Furthermore, community agencies and researchers can use these findings to create the necessary programming and tools to reduce HIV incidence for Black MSM.

## Appendix

Multimedia Appendix 1 PRISMA-ScR appendix.

Multimedia Appendix 2 Results of scoping review search: quantitative studies and meta-analysis, qualitative studies, and reports.

## Abbreviations

ACCHO: African and Caribbean Council on HIV or AIDS in Ontario
APAA: Africans in Partnership Against AIDS
Black CAP: Black Coalition for AIDS Prevention
CAAT: Committee for Accessible AIDS Treatment
GBTQ+: gay, bisexual, transgender, or queer
HBV: hepatitis B
HSV: herpes simplex virus
MSM: men who have sex with men
MSW: men who have sex with women
OHTN: Ontario HIV Treatment Network
PrEP: Preexposure prophylaxis
PRISMA-ScR: Preferred Reporting Items for Systematic Reviews and Meta-Analyses extension for Scoping Reviews
SBBI: sexual and blood-borne infection

## References

[1] McAlister FA, Cram P, Bell CM (2018). Comparing Canadian health care to that in other countries: looking beyond the headlines. CMAJ.

[2] Flag, Armed Forces and economy make Canadians proudest. Research Co.

[3] Waldron IRG (2010). The impact of inequality on health in Canada: a multi-dimensional framework. Divers Equal Health Care.

[4] Veenstra G, Patterson AC (2016). Black-White health inequalities in Canada. J Immigr Minor Health.

[5] Loignon C, Hudon C, Goulet É, Boyer S, De Laat M, Fournier N, Grabovschi C, Bush P (2015). Perceived barriers to healthcare for persons living in poverty in Quebec, Canada: the EQUIhealThY project. Int J Equity Health.

[6] Patel A, Dean J, Edge S, Wilson K, Ghassemi E (2019). Double Burden of Rural Migration in Canada? Considering the Social Determinants of Health Related to Immigrant Settlement Outside the Cosmopolis. Int J Environ Res Public Health.

[7] Magalhaes L, Carrasco C, Gastaldo D (2010). Undocumented migrants in Canada: a scope literature review on health, access to services, and working conditions. J Immigr Minor Health.

[8] Cloos P, Ndao EM, Aho J, Benoît Magalie, Fillol A, Munoz-Bertrand M, Ouimet MJ, Hanley J, Ridde V (2020). The negative self-perceived health of migrants with precarious status in Montreal, Canada: a cross-sectional study. PLoS One.

[9] McKinnon B, Yang S, Kramer MS, Bushnik T, Sheppard AJ, Kaufman JS (2016). Comparison of black-white disparities in preterm birth between Canada and the United States. CMAJ.

[10] Gahagan J, Subirana-Malaret M (2018). Improving pathways to primary health care among LGBTQ populations and health care providers: key findings from Nova Scotia, Canada. Int J Equity Health.

[11] Haddad N, Li JS, Totten S, McGuire M (2018). HIV in Canada-surveillance report, 2017. Can Commun Dis Rep.

[12] (2017). New HIV diagnoses in Ontario, 2017. Ontario HIV Epidemiology and Surveillance, Initiative.

[13] Quinn K, Bowleg L, Dickson-Gomez J (2019). "The fear of being Black plus the fear of being gay": the effects of intersectional stigma on PrEP use among young Black gay, bisexual, and other men who have sex with men. Soc Sci Med.

[14] Matthews DD, Herrick AL, Coulter RWS, Friedman MR, Mills TC, Eaton LA, Wilson PA, Stall RD, POWER Study Team (2016). Running backwards: consequences of current HIV incidence rates for the next generation of Black MSM in the United States. AIDS Behav.

[15] Dougan S, Elford J, Rice B, Brown AE, Sinka K, Evans BG, Gill ON, Fenton KA (2005). Epidemiology of HIV among black and minority ethnic men who have sex with men in England and Wales. Sex Transm Infect.

[16] Hickson F, Melendez-Torres GJ, Reid D, Weatherburn P (2017). HIV, sexual risk and ethnicity among gay and bisexual men in England: survey evidence for persisting health inequalities. Sex Transm Infect.

[17] Bourgeois AC, Edmunds M, Awan A, Jonah L, Varsaneux O, Siu W (2017). HIV in Canada-surveillance report, 2016. Can Commun Dis Rep.

[18] Haddad N, Robert A, Weeks A, Popovic N, Siu W, Archibald C (2019). HIV in Canada-surveillance report, 2018. Can Commun Dis Rep.

[19] (2017). NIAID editorial standards guide. National Institutes of Health.

[20] Quinn K, Voisin DR, Bouris A, Jaffe K, Kuhns L, Eavou R, Schneider J (2017). Multiple dimensions of stigma and health related factors among young Black men who have sex with men. AIDS Behav.

[21] Meyer IH (1995). Minority stress and mental health in gay men. J Health Soc Behav.

[22] Kelly JA, St Lawrence JS, Amirkhanian YA, DiFranceisco WJ, Anderson-Lamb M, Garcia LI, Nguyen MT (2013). Levels and predictors of HIV risk behavior among Black men who have sex with men. AIDS Educ Prev.

[23] Carter JW, Flores SA (2019). Improving the HIV prevention landscape to reduce disparities for Black MSM in the South. AIDS Behav.

[24] Wickham GRE (2017). Culturally appropriate health services for Black Canadians. J Student Sci Technol.

[25] Paradies Y, Ben J, Denson N, Elias A, Priest N, Pieterse A, Gupta A, Kelaher M, Gee G (2015). Racism as a determinant of health: a systematic review and meta-analysis. PLoS One.

[26] Brotman S, Ryan B, Jalbert Y, Rowe B (2002). The impact of coming out on health and health care access: the experiences of gay, lesbian, bisexual and two-spirit people. J Health Soc Policy.

[27] Chard AN, Finneran C, Sullivan PS, Stephenson R (2015). Experiences of homophobia among gay and bisexual men: results from a cross-sectional study in seven countries. Cult Health Sex.

[28] Wheeler DP, Fields SD, Beauchamp G, Chen YQ, Emel LM, Hightow-Weidman L, Hucks-Ortiz C, Kuo I, Lucas J, Magnus M, Mayer KH, Nelson LE, Hendrix CW, Piwowar-Manning E, Shoptaw S, Watkins P, Watson CC, Wilton L (2019). Pre-exposure prophylaxis initiation and adherence among Black men who have sex with men (MSM) in three US cities: results from the HPTN 073 study. J Int AIDS Soc.

[29] Brown AF, Ma GX, Miranda J, Eng E, Castille D, Brockie T, Jones P, Airhihenbuwa CO, Farhat T, Zhu L, Trinh-Shevrin C (2019). Structural interventions to reduce and eliminate health disparities. Am J Public Health.

[30] Hogg RS (2018). Understanding the HIV care continuum. Lancet HIV.

[31] Yehia BR, Stephens-Shields AJ, Fleishman JA, Berry SA, Agwu AL, Metlay JP, Moore RD, Mathews WC, Nijhawan A, Rutstein R, Gaur AH, Gebo KA (2015). The HIV care continuum: changes over time in retention in care and viral suppression. PLoS One.

[32] Djiadeu P, Yusuf A, Ongolo-Zogo C, Nguemo J, Odhiambo AJ, Mukandoli C, Lightfoot D, Mbuagbaw L, Nelson LE (2020). Barriers in accessing HIV care for Francophone African, Caribbean and Black people living with HIV in Canada: a scoping review. BMJ Open.

[33] Crenshaw K, Bartlett KT, Kennedy R (1989). Demarginalizing the intersection of race and sex: a Black feminist critique of antidiscrimination doctrine, feminist theory and antiracist politics. Feminist Legal Theory: Readings in Law and Gender.

[34] Nelson LE, Walker JJ, DuBois SN, Giwa S (2014). Your blues ain't like mine: considering integrative antiracism in HIV prevention research with Black men who have sex with men in Canada and the United States. Nurs Inq.

[35] Peterson J, Pearce PF, Ferguson LA, Langford CA (2017). Understanding scoping reviews: definition, purpose, and process. J Am Assoc Nurse Pract.

[36] Djiadeu P, Nur J, Mbuagbaw L, Giwa S, Whitfield D, Nelson LE (2021). HIV prevention and treatment interventions for Black men who have sex with men in Canada: a protocol for a scoping systematic review. BMJ Open.

[37] Ouzzani M, Hammady H, Fedorowicz Z, Elmagarmid A (2016). Rayyan-a web and mobile app for systematic reviews. Syst Rev.

[38] Viera AJ, Garrett JM (2005). Understanding interobserver agreement: the kappa statistic. Fam Med.

[39] Ahlm C, Wallin K, Lundkvist A, Elgh F, Juto P, Merza M, Tärnvik A (2000). Serologic evidence of Puumala virus infection in wild moose in northern Sweden. Am J Trop Med Hyg.

[40] Peters MDJ, Godfrey CM, Khalil H, McInerney P, Parker D, Soares CB (2015). Guidance for conducting systematic scoping reviews. Int J Evid Based Healthc.

[41] Bernardo WM (2017). PRISMA statement and PROSPERO. Int Braz J Urol.

[42] Stroup DF, Berlin JA, Morton SC, Olkin I, Williamson GD, Rennie D, Moher D, Becker BJ, Sipe TA, Thacker SB (2000). Meta-analysis of observational studies in epidemiology: a proposal for reporting. Meta-analysis of Observational Studies in Epidemiology (MOOSE) group. JAMA.

[43] (2012). Laboratory enhancement program; updated analyses: January 2009 to December 2012. Public Health Ontario.

[44] Nelson LE, Tharao W, Husbands W, Sa T, Zhang N, Kushwaha S, Absalom D, Kaul R (2019). The epidemiology of HIV and other sexually transmitted infections in African, Caribbean and Black men in Toronto, Canada. BMC Infect Dis.

[45] Lewis-Peart D (2007). Visibly hidden: rethinking Black MSM and HIV prevention. Rainbow Health Ontario.

[46] Djiadeu P, Smith MDR, Kushwaha S, Odhiambo AJ, Absalom D, Husbands W, Tharao W, Regan R, Sa T, Zhang N, Kaul R, Nelson LE (2020). Social, clinical, and behavioral determinants of HIV infection and HIV testing among Black men in Toronto, Ontario: a classification and regression tree analysis. J Int Assoc Provid AIDS Care.

[47] Liu J, Remis RS, Myers T, Husbands W (2009). Special report: ethnicity analysis in the lambda survey of men who have sex with men, Ontario 2007. Dalla Lana School of Public Health, University of Toronto and AIDS Committee of Toronto.

[48] George C, Makoroka L, Rourke SB, Adam BD, Remis RS, Husbands W, Read SE (2014). HIV testing by Black MSM in Toronto. SAGE Open.

[49] George C, Makoroka L, Husbands W, Adam BD, Remis R, Rourke S (2013). Sexual health determinants in Black men-who-have-sex-with-men living in Toronto, Canada. Ethn Inequalities Heal Soc Care.

[50] Husbands W, Makoroka L, George C, Adam B, Remis R, Rourke S, Beyene J (2006). Health, community and vulnerability to HIV among African, Caribbean and Black gay and bisexual men in Toronto. Interagency Coalition on AIDS and Development.

[51] Fonner VA, Dalglish SL, Kennedy CE, Baggaley R, O'Reilly KR, Koechlin FM, Rodolph M, Hodges-Mameletzis I, Grant RM (2016). Effectiveness and safety of oral HIV preexposure prophylaxis for all populations. AIDS.

[52] Tan DHS, Hull MW, Yoong D, Tremblay C, O'Byrne P, Thomas R, Kille J, Baril JG, Cox J, Giguere P, Harris M, Hughes C, MacPherson P, O'Donnell S, Reimer J, Singh A, Barrett L, Bogoch I, Jollimore J, Lambert G, Lebouche B, Metz G, Rogers T, Shafran S (2017). Canadian guideline on HIV pre-exposure prophylaxis and nonoccupational postexposure prophylaxis. Can Med Assoc J.

[53] Nelson LE, James L, Coleman T, Etowa J, Husbands W, Lofters A, Mitchell MO, Nguemo JD, Nnorom O, Oraka C, Rana J (2018). A recipe for increasing racial and gender disparities in HIV infection: a critical analysis of the Canadian guideline on pre-exposure prophylaxis and non-occupational post-exposure prophylaxis' responsiveness to the HIV epidemics among women and Black communities. Can J Hum Sex.

[54] Zhabokritsky A, Nelson LE, Tharao W, Husbands W, Sa T, Zhang N, Thomas-Pavanel J, Baidoobonso S, Kaul R (2019). Barriers to HIV pre-exposure prophylaxis among African, Caribbean and Black men in Toronto, Canada. PLoS One.

[55] Absalom D, Boyce T (2020). One of these things ain't like the other: exploring the HIV prevention needs of young adult Black same gender loving men—a report prepared for the Gay Men's Sexual Health Alliance. Gay Men's Sexual Health Alliance.

[56] Palangi A (2014). HIV health literacy for African, Caribbean, and Black men living with HIV/AIDS. University of Toronto (Canada).

[57] Crichlow W (2016). Buller Men and Batty Bwoys.

[58] Lee-Foon NK, Logie CH, Siddiqi A, Grace D (2020). “I just trust what Google says, it’s the Bible”: exploring young, Black gay and other men who have sex with men’s evaluation of sexual health information sources in Toronto, Canada. Can J Hum Sex.

[59] Lee-Foon NK, Logie CH, Siddiqi A, Grace D (2022). Exploring young Black gay, bisexual and other men who have sex with men's PrEP knowledge in Toronto, Ontario, Canada. Cult Health Sex.

[60] Millett GA, Peterson JL, Flores SA, Hart TA, Jeffries WL, Wilson PA, Rourke SB, Heilig CM, Elford J, Fenton KA, Remis RS (2012). Comparisons of disparities and risks of HIV infection in Black and other men who have sex with men in Canada, UK, and USA: a meta-analysis. Lancet.

[61] George C, Adam BD, Read SE, Husbands WC, Remis RS, Makoroka L, Rourke SB (2012). The MaBwana Black men's study: community and belonging in the lives of African, Caribbean and other Black gay men in Toronto. Cult Health Sex.

[62] Husbands W, Makoroka L, Walcott R, Adam BD, George C, Remis RS, Rourke SB (2013). Black gay men as sexual subjects: race, racialisation and the social relations of sex among Black gay men in Toronto. Cult Health Sex.

[63] Zelaya DG, Guy AA, Surace A, Mastroleo NR, Pantalone DW, Monti PM, Mayer KH, Kahler CW (2022). Modeling the impact of race, socioeconomic status, discrimination and cognitive appraisal on mental health concerns among heavy drinking HIV+ cisgender MSM. AIDS Behav.

[64] (2007). Black coalition for AIDS prevention annual report, 2006. Black Coalition for AIDS Prevention.

[65] (2020). Black coalition for AIDS prevention (Black CAP) annual report 2019/20. Black Coalition for AIDS Prevention.

[66] (2015). Black coalition for AIDS prevention annual report, 2014/15. Black Coalition for AIDS Prevention.

[67] Dulai J, Le D, Ferlatte O, Marchand R, Trussler T Sex now across Canada: highlights from the sex now survey by province. Programme National De Mentorat Sur Le Vih Et Les Hépatites.

[68] Sharma M, Wilton J, Senn H, Fowler S, Tan DHS (2014). Preparing for PrEP: perceptions and readiness of Canadian physicians for the implementation of HIV pre-exposure prophylaxis. PLoS One.

[69] Mulqueeny DM, Nkabini SM, Pokiya MH (2021). Mapping evidence of depression in HIV-seropositive MSM in sub-Saharan Africa: a scoping review protocol. Syst Rev.

[70] Tran BX, Ho RCM, Ho CSH, Latkin CA, Phan HT, Ha GH, Vu GT, Ying J, Zhang MWB (2019). Depression among patients with HIV/AIDS: research development and effective interventions (GAPRESEARCH). Int J Environ Res Public Health.

[71] Levesque JF, Harris MF, Russell G (2013). Patient-centred access to health care: conceptualising access at the interface of health systems and populations. Int J Equity Health.

[72] National Academies of Sciences, Engineering, and Medicine, Health and Medicine Division, Committee on Health Care Utilization and Adults with Disabilities, Board on Health Care Services (2018). Factors that affect health-care utilization. Health-Care Utilization as a Proxy in Disability Determination.

[73] Tao J, Vermund SH, Lu H, Ruan Y, Shepherd BE, Kipp AM, Amico KR, Zhang X, Shao Y, Qian HZ (2017). Impact of depression and anxiety on initiation of antiretroviral therapy among men who have sex with men with newly diagnosed HIV infections in China. AIDS Patient Care STDS.

[74] Manicaland Centre for Public Health Research (2017). HIV prevention cascades.

[75] Elopre L, Ott C, Lambert CC, Amico KR, Sullivan PS, Marrazzo J, Mugavero MJ, Turan JM (2021). Missed prevention opportunities: why young, Black MSM with recent HIV diagnosis did not access HIV pre-exposure prophylaxis services. AIDS Behav.

[76] Babel RA, Wang P, Alessi EJ, Raymond HF, Wei C (2021). Stigma, HIV risk, and access to HIV prevention and treatment services among men who have sex with men (MSM) in the United States: a scoping review. AIDS Behav.

[77] Frye V, Nandi V, Paige MQ, McCrossin J, Lucy D, Gwadz M, Sullivan PS, Hoover DR, Wilton L (2021). TRUST: assessing the efficacy of an intervention to increase HIV self-testing among young black men who have sex with men (MSM) and transwomen. AIDS Behav.

[78] Scott HM, Pollack L, Rebchook GM, Huebner DM, Peterson J, Kegeles SM (2014). Peer social support is associated with recent HIV testing among young black men who have sex with men. AIDS Behav.

[79] Washington TA, Applewhite S, Glenn W (2017). Using Facebook as a platform to direct young Black men who have sex with men to a video-based HIV testing intervention: a feasibility study. Urban Soc Work.

[80] Jones KT, Gray P, Whiteside YO, Wang T, Bost D, Dunbar E, Foust E, Johnson WD (2008). Evaluation of an HIV prevention intervention adapted for Black men who have sex with men. Am J Public Health.

[81] Liang TS, Erbelding E, Jacob CA, Wicker H, Christmyer C, Brunson S, Richardson D, Ellen JM (2005). Rapid HIV testing of clients of a mobile STD/HIV clinic. AIDS Patient Care STDS.

[82] Gardner LI, Marks G, Shahani L, Giordano TP, Wilson TE, Drainoni ML, Keruly JC, Batey DS, Metsch LR (2016). Assessing efficacy of a retention-in-care intervention among HIV patients with depression, anxiety, heavy alcohol consumption and illicit drug use. AIDS.

[83] Genberg BL, Shangani S, Sabatino K, Rachlis B, Wachira J, Braitstein P, Operario D (2016). Improving engagement in the HIV care cascade: a systematic review of interventions involving people living with HIV/AIDS as peers. AIDS Behav.

[84] Hightow-Weidman LB, Pike E, Fowler B, Matthews DM, Kibe J, McCoy R, Adimora AA (2012). HealthMpowerment.org: feasibility and acceptability of delivering an internet intervention to young Black men who have sex with men. AIDS Care.

[85] Higa DH, Marks G, Crepaz N, Liau A, Lyles CM (2012). Interventions to improve retention in HIV primary care: a systematic review of U.S. studies. Curr HIV/AIDS Rep.

[86] Lancaster KE, Miller WC, Kiriazova T, Sarasvita R, Bui Q, Ha TV, Dumchev K, Susami H, Hamilton EL, Rose S, Hershow RB, Go VF, Metzger D, Hoffman IF, Latkin CA (2019). Designing an individually tailored multilevel intervention to increase engagement in HIV and substance use treatment among people who inject drugs with HIV: HPTN 074. AIDS Educ Prev.

[87] Wingood GM, Lambert D, Renfro T, Ali M, DiClemente RJ (2019). A multilevel intervention with African American churches to enhance adoption of point-of-care HIV and diabetes testing, 2014-2018. Am J Public Health.

[88] Kegeles SM, Rebchook G, Tebbetts S, Arnold E, TRIP Team (2015). Facilitators and barriers to effective scale-up of an evidence-based multilevel HIV prevention intervention. Implement Sci.

