# Supplementary material for: HIV Prevention and Treatment Interventions for Black Men Who Have Sex With Men in Canada: Scoping Systematic Review
Source: JMIR Public Health Surveill. 2024 Jan 18;10:e40493. doi: 10.2196/40493 (PMC10835596; doi:10.2196/40493)
Supplement: Multimedia Appendix 2 [file publichealth_v10i1e40493_app2.docx]

**Table I: Results of scoping review search: quantitative studies and meta-analysis, qualitative studies, and reports**

| **Authors** | **Year** | **Study Location** | **Design** | **Study Sample** | **Outcomes/Themes** | **Key findings** |
| --- | --- | --- | --- | --- | --- | --- |
| Liu et al. [43] | 2007 | Ontario | Cross-sectional | 2,438 MSM | HIV and STI epidemiology; testing history; risk behaviour; MSM sexual health and sexual behaviour | 1. African/Caribbean MSM had higher HIV prevalence than some other ethnic groups (11.8%, all p<0.05). 2. 50.0% of Africa/Caribbean MSM in Toronto did not know they were HIV-positive. 3. In Toronto, about 25-30% of MSM had at least 10 casual partners, except African/Caribbean MSM at 14.1%. 4. In Toronto,.6% of African/Caribbean MSM had unprotected receptive anal sex with a casual partner or HIV-positive regular partner or unknown HIV status regular partner during the previous six months. |
| Sullivan & Remis [44] | 2012 | Ontario | Cross-sectional | 2335 MSM | HIV testing history; HIV epidemiology | 1. Black MSM HIV diagnoses increased from 2009 – 2012, while they decreased for White MSM |
| George et al. [45] | 2013 | Toronto | Cross-sectional | 168 Black MSM | HIV/STI risk factor analysis | 1. 22% of HIV tests for Black MSM are positive, of which 25% had disclosed their status 2. African-born (60%) or Caribbean-born (54.8%) men were more likely to know someone who died from HIV/AIDS-related illness, compared to Canadian-born men (28%) 3. 55% of men reported seeing ACCHO’s ‘Keep It Alive’ campaign and 43% reported seeing ‘Be Real,’ but awareness of most local campaigns remain limited 4. Awareness of campaigns was not associated with consistent condom use |
| George et al. [46] | 2014 | Toronto | Cross-sectional | 168 Black MSM | Investigation of significant-influencing factors to HIV testing | 1. Canadian-born (21.6%) men were more likely to not be tested for HIV, compared to African-born (7.0%) or Caribbean (7.0%) men 2. Younger men were less likely to test for HIV than older men 3. 49% of participants received HIV tests with their family doctor’s office 4. The most common reasons to not test for HIV were low-risk perception and reliance on ‘safe sex’ |
| Nelson et al. [47] | 2019 | Toronto | Cross-sectional | 487 Black men | HIV/STI diagnosis epidemiology; intra-racial comparison between Black MSM and Black men who only have sex with women (MSW) | 1. HIV prevalence among MSM (38%) was higher than MSW (3%), as well as the general Black population in Ontario and MSM in Ontario 2. A higher proportion of syphilis diagnoses among MSM than MSW |
| Zhabokritsky et al. [48] | 2019 | Toronto | Cross-sectional | 424 Black men | ACB men PrEP use; risk perception | 1. A higher proportion of syphilis diagnoses among MSM than MSW 2. Black MSM who were young or born in Canada were less likely to accept PrEP 3. Black MSM were more likely to accept PrEP than Black MSW 4. Black MSM were not able to accurately describe their risk of HIV transmission |
| Djiadeu et al. [49] | 2020 | Toronto | Cross-sectional | 487 Black men | HIV infection risk factor analysis; determining factors for HIV testing for Black men | 1. HIV infection rate was higher for Black MSM (42.11%) and MSM who also have sex with women (36.59%), compared to MSW (2.91%) 2. The number of male partners is the best sole predictor of HIV infection |
| Husbands et al. [50] | 2006 | Toronto | Cross-sectional, qualitative | 175 Black MSM | Risk factors for HIV; Black MSM HIV knowledge; healthcare access | 1. 39.4% of men living with HIV had sex with HIV-negative men, with only 61.5% using condoms all the time 2. 36.4% of men with HIV had sex with men of unknown status, with only 41.7% reported using condoms consistently 3. In-access to prevention methods mentioned as one of the top reasons for not using protection 4. ACCHO and Ontario Gay Men’s Strategy campaigns made to increase HIV literacy, HIV testing, and reduce HIV stigma through ads and posters |
| Millett et al. [51] | 2012 | Canada, UK, USA | Meta-analysis | 7 studies | International comparisons of HIV risk factors | 1. Black MSM showed no difference in HIV or STI diagnosis 2. Black MSM were more likely to have income as a barrier to accessing HIV interventions |
| Crichlow [52] | 2004 | Toronto, Halifax | Qualitative | 19 Black MSM | Racialization and heterosexism; Black MSM community support; stigma and discrimination; critical analysis of Black MSM behaviour and experience | 1. Black MSM experience racism and heterosexism in Canada at large and within Black and gay communities 2. Community stigma contributes to the risk of depression and poor mental health outcomes 3. Black MSM experience discrimination within healthcare organizations while receiving HIV care 4. Black MSM require HIV literacy and support to reduce HIV risk |
| George et al. [53] | 2012 | Toronto | Qualitative | 175 Black MSM | Community-support and community-building; racialized and sexual community analysis; foundation for culturally responsive services | 1. Black MSM are marginalized within gay communities 2. Black MSM face difficulties in receiving support from their ethnic communities 3. Black MSM experience HIV stigma, even when they are not HIV-infected 4. Services and programming for Black gay men need to address the complex interactions between their intersecting stigmas |
| Gillis & Palangi  [54] | 2015 | Toronto | Qualitative | 8 Black men | HIV health literacy; accessibility to and usage of HIV care services; barriers and facilitators to HIV care | 1. Black migrant MSM are not linked to care by immigration doctors 2. Emotional and mental health counseling is not available to Black migrant MSM 3. Black migrant MSM experience gaps in care due to immigration status and complex immigration laws and policies 4. Black migrant MSM living with HIV experience HIV stigma and homophobia, which reduces their ability to disclose their HIV status 5. Community stigma and hypermasculinity act as a barrier to HIV-care |
| Lee-Foon et al.  [55] | 2020 | Toronto | Qualitative | 22 Black MSM | Sexual health literacy; PrEP uptake for young Black MSM | 1. Young Black MSM have three main sources for sexual health information: friends, the Internet, and healthcare providers 2. Outdated sexual education and homophobic stigma reduce young Black MSM sexual health literacy 3. Young Black MSM rely heavily on healthcare providers for linkage to PrEP, which would be an important point for interventions |
| Lee-Foon et al. [56] | 2020 | Toronto | Qualitative | 22 Black MSM | Black MSM knowledge sourcing of PrEP | 1. Outdated sexual education and homophobic stigma reduce PrEP knowledge and motivation 2. Interventions need to consider socio-structural factors and their impact on PrEP knowledge for young Black MSM |
| Absalom & Boyce [57] | 2020 | Toronto. Ottawa | Report [Qualitative, focus groups] | 21 Black MSM | HIV Preventative services; Healthcare access; HIV service provision quality | 1. Black MSM are not linked to HIV preventative interventions through their providers 2. Black MSM are discriminated against due to their racial and sexual identities in HIV services 3. Structural disparities reduce access to HIV interventions |
| Black Coalition for AIDS Prevention (Black CAP) [58] | 2007 | Toronto | Organizational Annual Report | | Support services; health promotion efforts | 1. Facilitated HIV awareness through outreach programs |
| Black Coalition for AIDS Prevention (Black CAP) [59] | 2015 | Toronto | Organizational Annual Report | | Support services; health promotion efforts | 1. MSM outreach program to increase HIV awareness, HIV testing, and HIV service access |
| Black Coalition for AIDS Prevention [60] (Black CAP) | 2020 | Toronto | Organizational Annual Report | | Support services; health promotion efforts | 1. Created partnerships with local sexual health clinics 2. 3MV program to provide culturally responsive HIV education and increase PrEP knowledge and access 3. Created online resources for Black MSM to increase access to sexual health services 4. Linked to support services to increase food access, financial security, and stable employment |
| Lewis-Peart [61] | 2007 | Toronto | Organizational Report | | HIV healthcare delivery; prevention service assessment; HIV/STI risk factor analysis; guidelines for culturally appropriate care; sexual health education for Black MSM; stigma reduction | 1. Sexual identities need to be recognized in prevention messaging for Black MSM 2. Comprehensive HIV and other SBBI testing and prevention programs are needed to reduce transmission for young Black MSM 3. Social constructs (e.g. masculinity) need to be addressed in intervention programming 4. There is a lack of interventions for Black MSM in Toronto |
